# RETRACTION: A Schiff Base‐Derived Copper (II) Complex Is a Potent Inducer of Apoptosis in Colon Cancer Cells by Activating the Intrinsic Pathway.

**DOI:** 10.1155/tswj/9846859

**Published:** 2026-06-02

**Authors:** The Scientific World Journal

RETRACTION: M. Hajrezaie, M. Paydar, S. Z. Moghadamtousi, P. Hassandarvish, N. S. Gwaram, M. Zahedifard, E. Rouhollahi, H. Karimian, C. Y. Looi, H. M. Ali, N. A. Majid, M. A. Abdulla, “A Schiff Base‐Derived Copper (II) Complex Is a Potent Inducer of Apoptosis in Colon Cancer Cells by Activating the Intrinsic Pathway,” *The Scientific World Journal*, 2014, 540463, 10.1155/2014/540463

The above article, published online on 05 March 2014 in Wiley Online Library (http://wileyonlinelibrary.com), has been retracted by agreement between the Academic Editor, Thomas Efferth; and John Wiley & Sons Ltd. The retraction has been agreed due to concerns identified with Figure 3a on PubPeer [1]. Further investigation by the publisher identified additional concerns with Figure 4a. A summary of the concerns is as follows:

• Figure 3b is identical to Figure 11a in [2]
• The panels in Figure 4a appear identical to Figure 2a of [3]

Author Pouya Hassandarvish responded to confirm that the error is in the image presented in [2] (which was published by some of the same authors), however, no authors responded to provide any original images or further explanation. After assessment by the Editorial Board of all concerns raised and the author’s response, the results and conclusions presented can no longer be considered reliable and the article is therefore retracted.

The authors did not respond to the retraction.
